# Serum IL-33 but not ST2 level is elevated in intermittent allergic rhinitis and is a marker of the disease severity

**DOI:** 10.1007/s00011-012-0443-9

**Published:** 2012-02-15

**Authors:** Joanna Glück, Barbara Rymarczyk, Barbara Rogala

**Affiliations:** Department of Internal Diseases, Allergology and Clinical Immunology, Medical University of Silesia, ul. Ceglana 35, 40-952 Katowice, Poland

**Keywords:** ST2, IL-33, Intermittent allergic rhinitis, Bronchial asthma, IgE, Total nasal symptom score

## Abstract

**Background:**

Th2 cells play an important role in intermittent allergic rhinitis (IAR). Interleukin (IL)-33 stimulates the production of Th2-associated cytokines. IL-33 binds to ST2 receptor which is highly expressed on mast cells and selectively on Th2 cells. IL-33 and ST2 might be involved in the Th2-mediated immune response.

**Objective:**

We analyzed the serum level of IL-33 and its receptor ST2 in patients with IAR sensitive to grass and/or tree pollen to assess if the serum level of IL-33 and/or ST2 may be a marker of the disease severity.

**Methods:**

IL-33, ST2 and total immunoglobulin (Ig) E were measured in sera of patients with IAR sensitive to birch and/or grass pollen and in patients with controlled bronchial asthma and in non-allergic controls. IAR severity was assessed by total nasal symptom score.

**Results:**

Serum levels of IL-33 in patients with IAR were comparable with patients with bronchial asthma and were significantly higher in patients with IAR (*P* = 0.0035) and in patients with bronchial asthma (*P* = 0.008) than in controls. Serum levels of IL-33 correlated with disease severity.

**Conclusion:**

Elevated level of IL-33 in sera of patients with IAR sensitive to tree and/or grass pollen and the correlation of IL-33 with the disease severity suggest that IL-33 is involved in the pathogenesis of intermittent allergic rhinitis.

## Introduction

Allergic diseases are thought to be Th2 cell-mediated diseases. Th2 cells produce cytokines, such as interleukin (IL)-4, IL-5 and IL-13. Recently it has been shown that a novel cytokine, IL-33, is also involved in the Th2-mediated immune response and stimulates the production of Th2-associated cytokines. IL-33 is also a chemoattractant for human Th2 cells [1]. IL-33 is produced by mast cells after immunoglobulin (Ig) E-mediated activation and is able to trigger mast cells to release proinflammatory cytokines in vitro [2–5].

IL-33 is a member of the IL-1 family of cytokines and binds to two receptors: ST2 (IL-1R1) and IL-1 receptor accessory protein (IL-1RAP). There are two isoforms of ST2 proteins: ST2L, a transmembrane form, and soluble ST2 (sST2), a secreted form that can serve as a decoy receptor of IL-33. ST2 is highly expressed on mast cells and selectively on Th2 cells [6–8]. High levels of sST2 have been found in the sera of adults and children with acute asthma [9, 10]. The IL-33/ST2 signalling pathway activates airway eosinophils that exacerbate airway inflammation in an autocrine and paracrine manner [11]. This pathway is critical for the progression of IgE-dependent inflammation [2]. Mutations in the gene for IL1RL1 (ST2) have been linked to atopic dermatitis and asthma [12, 13].

IL-33 induces anaphylactic shock in mice and is markedly elevated in the serum of patients during anaphylactic shock and in atopic human tissue [14]. The role of IL-33 in bronchial asthma has been extensively studied [15, 16]. However, there are less data on the role of IL-33 and its receptor ST2 in intermittent allergic rhinitis. The serum level of IL-33 was found to be higher in patients with Japanese cedar pollinosis [17], However, it has not been studied in intermittent allergic rhinitis in patients allergic to other allergens. Moreover, to date the serum level of ST2 has not been analyzed in allergic rhinitis. We therefore decided to analyze the serum level of IL-33 and the soluble form of its receptor ST2 in patients with intermittent allergic rhinitis sensitive to grass and/or tree pollen and to compare with results in bronchial asthma patients and healthy controls. Another aim of the study was to assess if the serum level of IL-33 and/or ST2 may be a marker of the disease severity.

## Methods

Twenty-six patients (14 women, median age 32 years [25–43]; group IAR) with intermittent allergic rhinitis sensitive to grass and/or tree pollen were included in the study. Two control groups were selected. The former involved 21 patients (12 women, median age 36 years [27–48]) with controlled bronchial asthma (group AST) and the latter 27 healthy subjects (15 women, median age 40 years [22–52], group CON) with no signs of allergic diseases nor any inflammatory diseases. The patients were otherwise healthy, which means that apart from allergic disease they did not suffered from any other serious chronic diseases, such as cardiac, renal or gastrointestinal diseases, and from any acute inflammatory disease. These conditions belonged to the exclusion criteria.

The diagnosis of intermittent allergic rhinitis was based on clinical history and skin prick test results. Rhinitis was stated as intermittent according to ARIA criteria [18]. In all cases, the serum levels of allergen-specific IgE (as-IgE) against the most clinically important allergens were estimated to confirm the diagnosis. The patients with intermittent allergic rhinitis were evaluated in the symptomatic phase of the disease, during the birch pollen season (end of April, May) or grass pollen season (end of May until end of July). The diagnosis of controlled bronchial asthma was based on clinical history, physical findings and lung function test results [19]. Patients suffering only from seasonal asthma were not included into the study.

None of the patients had used systemic corticosteroids within the last 3 months. The patients with intermittent allergic rhinitis were allowed to use topical intranasal steroids and/or nasal decongestants. The patients with bronchial asthma were allowed to use inhalant glucocorticosteroids and short or/and long-acting β_2_-mimetics. All patients gave informed consent. The study was approved by the Local Ethics Committee at the Medical University of Silesia.

### Symptom score in intermittent allergic rhinitis

Patients with intermittent allergic rhinitis evaluated nasal symptoms using a 4-point scale (Total Nasal Symptom Score; TNSS) comprising four items: nasal congestion, nasal itching, sneezing and rhinorrhea, assessed on a 0–3 category scale.

### Skin prick tests

Skin prick tests (SPT) were performed according to the EAACI guidelines [20].

### Measurement of serum levels of ST2, IL-33, total and allergen-specific IgE

Commercial enzyme-linked immunosorbent assays were used to measure serum levels of ST2L/IL-1 R4 (R&D Systems) and IL-33 (GenWay). The assay was performed using the protocols recommended by the manufacturers. Total IgE and as-IgE levels were determined by using ELISA (Allergopharma). Levels of specific IgE > 0.35 (≥class 1) were considered positive.

### Statistical analysis

Results are expressed as median values with interquartile ranges. Nonparametric tests were used (Mann–Whitney *U* rank sum test and Spearman’s correlation test). All analyses were performed with a software package (Quick Statistica Pl 5.1). *P* values less than 0.05 were considered significant.

## Results

**Fig. 1:**
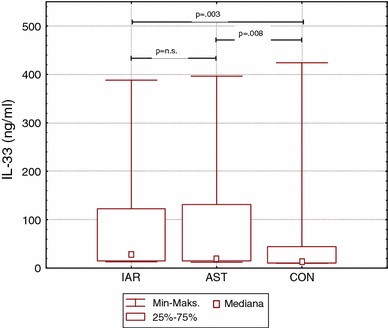
Median values, interquartile and total range of IL-33 serum levels in intermittent allergic rhinitis and asthma patients and in controls. *IAR* intermittent allergic rhinitis group, *AST* asthma group, *CON* control group

Serum levels of IL-33 were significantly higher in IAR and AST groups than in CON group (28.5 ng/ml [15–123], 19 ng/ml [15–132], 14 ng/ml [11–44], respectively) and did not differ between IAR and AST groups (Fig. 1). Serum levels of ST2 and total IgE were comparable in all groups. Detailed data are shown in Table 1.

**Table 1:** Serum levels of IL-33, ST2, total and allergen-specific IgE (median and interquartile ranges)

	Intermittent allergic rhinitic patients	Asthma patients	Controls	*P*
IL-33 (ng/ml)	28.5 (15–123)	19 (15–132)	14 (11–44)	0.0035 IAR versus CON, 0.008 AST versus CON
ST2L/IL-1 R4 (ng/ml)	518.5 (293–866)	720 (400–1,090)	590 (457.5–794.5)	n.s.
Total IgE (kU/L)	65 (40–160)	49 (30–97)	42 (14–88)	n.s.
As-IgE (kU/L)	9.08 (2.84–10.1)	n.a.	n.a.	n.a.

*n.s.* non-significant; *n.a.* not applicable

*IAR* intermittent allergic rhinitis group, *AST* asthma group, *CON* control group

Median TNSS in the IAR group was 8 points (interquartile range: 7–9, range 4–11).

Serum levels of IL-33 significantly correlated with TNSS (rs = 0.54; *P* = 0.004). The correlations between serum levels of IL-33 and total IgE and the clinically most important as-IgE level were non-significant. Correlations between ST2 serum level and TNSS, total IgE and as-IgE were non-significant (data not shown).

## Discussion

In the present study we found that a serum level of IL-33 is a marker of Th2-mediated allergic diseases, such as intermittent allergic rhinitis and, moreover, IL-33 is a marker of the intermittent allergic rhinitis severity. The serum level of IL-33 was comparable with results found in the asthmatic group. We failed to show that the form of IL-33 receptor, i.e. ST2, is elevated in serum of patients suffering from intermittent allergic rhinitis.

Intermittent allergic rhinitis and bronchial asthma belong to the group of allergic diseases mediated by Th2-type cytokines, such as IL-4, IL-5, IL-13. These cytokines are secreted by a subpopulation of T helper cells, called Th2 cells. It has been recently shown that IL-33 stimulates Th2 cells to secrete cytokines and is also a chemoattractant for these cells [1]. Furthermore, IL-33 seems increasingly to be an important cytokine in many inflammatory diseases, such as inflammatory bowel disease, rheumatologic disorders or central nervous system inflammation. On the other hand, IL-33 has protective effects in cardiovascular diseases, diabetes mellitus type 2 and obesity [21, 22]. IL-33 mediates its biological effects via interaction with the two receptors, ST2 (IL-1RL1) and IL-1 receptor accessory protein. Both of them are expressed on many immune cells, among them Th2 cells [6–8]. IL-33 acts also on other cells important in allergic reactions, e.g. mast cells. IL-33 activates mast cell degranulation through phospholipase D1 and sphingosine kinase-1 [14]. However, the presence of preformed IgE is critical for IL-33-induced mast cell degranulation.

The role of IL-33 has been shown in some allergic diseases, such as anaphylactic shock, atopic dermatitis or bronchial asthma [9, 10, 12, 14]. The serum level of IL-33 was studied in a large group of patients suffering from allergic rhinitis sensitive to Japanese cedar [17]. The serum level of the cytokine was higher than in controls and this result is in accordance with our findings. Moreover, a positive correlation was found between IL-33 polymorphism and Japanese cedar pollinosis. The role of IL-33 was also confirmed in the pathogenesis of allergic conjunctivitis using an experimental animal model of sensitization to ragweed pollen [23]. Matsuba-Kitamura et’al. found that IL-33 significantly increases the capacity of T cells to produce Th2-type cytokines, increases the infiltration of cervical lymph nodes by eosinophils and Th2 cells, and that conjunctival tissues constitutively express biologically active IL-33, suggesting that IL-33 might play a crucial role in the induction and augmentation of allergic conjunctivitis.

Interestingly, it has been shown recently that anti-IL-33 antibody has a therapeutic potential for experimental allergic rhinitis in a murine model of nasal allergy [24]. Anti-IL-33 antibodies relieved nasal symptoms assessed on the basis of nose-scratching events, and reduced the number of eosinophils in bronchoalveolar lavage (BAL) and in nasal cavity infiltration and Th2-type cytokines levels in BAL. Thus one can assume that IL-33 may be a potential therapeutic target against allergy in humans too.

Taken together, the results of our study and previously published papers confirm the role of IL-33 in Th2-mediated diseases, such as intermittent allergic rhinitis.

In our study we failed to show the role of ST2 receptor in intermittent allergic rhinitis based on the estimation of serum level of this receptor. Previous studies yielded the evidence of the role of innate immune response in activation and differentiation of Th2 cells. ST2, being a receptor for IL-33, constitutes part of this pathway [25]. The role of the soluble form of ST2 was also confirmed in allergic asthma [26]. In our study the serum level of ST2 was comparable both with healthy controls and with asthmatic patients. This can be explained by the fact that the intensity of systemic inflammation is much lower in intermittent allergic rhinitis than is found in other allergic diseases, such as bronchial asthma. It should be noted that in previously published papers the serum level of ST2 was only slightly higher in the asymptomatic phase of bronchial asthma and rose during an asthma attack in adults or in acute asthma in children [9, 10]. The level of ST2 should therefore be studied in nasal lavage or in nasal mucosa in further projects.

In conclusion, we found elevated levels of IL-33 in sera of patients with intermittent allergic rhinitis sensitive to tree and/or grass pollen, and that the serum level of IL-33 correlated with the disease severity. Our results suggest that IL-33 is involved in the pathogenesis of intermittent allergic rhinitis. The results obtained may provide new insight into the pathophysiology of the disease and into novel therapeutic targets.
